# Muscle hypertrophy is associated with increases in proteasome activity that is independent of MuRF1 and MAFbx expression

**DOI:** 10.3389/fphys.2014.00069

**Published:** 2014-02-21

**Authors:** Leslie M. Baehr, Matthew Tunzi, Sue C. Bodine

**Affiliations:** ^1^Department of Physiology and Membrane Biology, University of CaliforniaDavis, Davis, CA, USA; ^2^Department of Neurobiology, Physiology, and Behavior, University of CaliforniaDavis, Davis, CA, USA

**Keywords:** ubiquitin proteasome system, protein degradation, puromycin, functional overload, forkhead transcription factors

## Abstract

The regulation of skeletal muscle mass depends on the balance between protein synthesis and degradation. The role of protein degradation and in particular, the ubiquitin proteasome system, and increased expression of the E3 ubiquitin ligases, MuRF1 and MAFbx/atrogin-1, in the regulation of muscle size in response to growth stimuli is unclear. Thus, the aim of this study was to measure both proteasome activity and protein synthesis in mice over a 14-day period of chronic loading using the functional overload (FO) model. Further, the importance of MuRF1 and MAFbx expression in regulating muscle hypertrophy was examined by measuring muscle growth in response to FO in mice with a null deletion (KO) of either MuRF1 or MAFbx. In wild type (WT) mice, the increase in muscle mass correlated with significant increases (2-fold) in protein synthesis at 7 and 14 days. Interestingly, proteasome activity significantly increased in WT mice after one day, and continued to increase, peaking at 7 days following FO. The increase in proteasome activity was correlated with increases in the expression of the Forkhead transcription factors, FOXO1 and FOXO3a, which increased after both MuRF1 and MAFbx increased and returned to baseline. As in WT mice, hypertrophy in the MuRF1 and MAFbx KO mice was associated with significant increases in proteasome activity after 14 days of FO. The increase in plantaris mass was similar between the WT and MuRF1 KO mice following FO, however, muscle growth was significantly reduced in female MAFbx KO mice. Collectively, these results indicate that muscle hypertrophy is associated with increases in both protein synthesis and degradation. Further, MuRF1 or MAFbx expression is not required to increase proteasome activity following increased loading, however, MAFbx expression may be required for proper growth/remodeling of muscle in response to increase loading.

## Introduction

Skeletal muscle is a highly plastic tissue that modifies its size through the regulation of signaling pathways that control protein synthesis and protein degradation. In response to increases in mechanical loading, muscle hypertrophy, or an increase in muscle size, occurs as the result of a net increase in protein synthesis relative to degradation. It has been well demonstrated that the Akt/mTOR signaling pathway is a major regulator of muscle growth, as activation of S6K1, eIF4E, and eIF2B stimulate mRNA translation and ultimately lead to increases in protein synthesis (Bodine et al., 2001b; Rommel et al., 2001; Kubica et al., 2008). In addition, recent work has revealed that both beta adrenergic signaling (Minetti et al., 2011) and bone morphogenetic protein (BMP) signaling (Sartori et al., 2013) can regulate muscle mass and promote skeletal muscle hypertrophy. What is less understood is the role of protein degradation in the remodeling process that occurs in response to loading and leads to an increase in fiber cross-sectional area.

Increases in protein degradation are generally associated with the loss of muscle mass, i.e., atrophy, and occur in response to decreased loading, inactivity, and a variety of pathological conditions. In skeletal muscle, the ubiquitin proteasome system (UPS) is responsible for the majority of protein degradation (Rock et al., 1994), although cathepsins, calpains, caspase-3, and autophagy are also involved in the breakdown of muscle proteins (Du et al., 2004; Tisdale, 2005). Associated with muscle atrophy and the increase in protein degradation is the rapid and sustained increase in MuRF1 and MAFbx/atrogin-1 expression, two muscle-specific E3 ubiquitin ligases thought to target specific proteins for degradation by the 26S proteasome (Bodine et al., 2001a). Deletion of MuRF1 or MAFbx has been shown to spare muscle mass in a variety of atrophy-inducing conditions (Bodine et al., 2001a; Labeit et al., 2010; Baehr et al., 2011), however, the role of MuRF1 and MAFbx, as well as the UPS, in regulating increases in muscle fiber size is less clear.

A few studies have reported increases in MuRF1 and MAFbx expression following an acute bout of resistance exercise in humans, however, no studies have made concurrent measurements of protein synthesis and UPS activity following chronic mechanical loading (Yang et al., 2006; Louis et al., 2007; Marino et al., 2008). Thus, the aim of this study was to examine both protein synthesis and proteasome activity, along with MuRF1 and MAFbx expression in mice over 14 days of chronic loading using the functional overload (FO) model. Furthermore, although we have recently shown that muscle growth is not impaired in young or old MuRF1 KO mice (Hwee et al., 2013), it remains to be seen whether growth is affected by the loss of MAFbx, and whether the loss of MuRF1 or MAFbx depresses proteasome activity under anabolic conditions.

## Materials and methods

### Animals

Forty three month old male C57BL/6 wild type (WT) mice and 110 9 month old male and female MuRF1 and MAFbx null (KO) mice were used for this study. The WT mice were purchased from Jackson Laboratories and the MuRF1 (*n* = 60) and MAFbx (*n* = 50) null mice were generated from a breeding colony maintained by the UCD Mouse Biology Program in a mouse barrier facility. To induce hypertrophy of the plantaris muscle, mice were subjected to bilateral functional overload. Mice were anaesthetized with 2–3% inhaled isoflurane and using aseptic technique, the ankle extensor muscles and Achilles tendon were exposed by making a small incision to the posterior lower limb. The entire soleus and over half of the medial and lateral gastrocnemius muscles were removed from each hindlimb without damaging the plantaris neural-vascular supply. The wound was irrigated with sterile saline and the incision was closed with subcuticular sutures. Mice were given an analgesic (buprenorphine, 0.1 mg/kg) immediately following the surgery and returned to their cage once they recovered.

At 1, 3, 7, and 14 days post-surgery, the WT animals were anesthetized with 2–3% inhaled isoflurane and the plantaris muscles were removed, weighed, frozen in liquid nitrogen, and stored at −80°C for future analysis. For the MuRF1 and MAFbx KO mice, the plantaris muscles were removed and weighed following 14 days of FO. The right plantaris muscle was pinned on cork at a length approximating L_o_ and frozen in isopentane cooled in liquid nitrogen for histological analysis while the left plantaris muscle was frozen in liquid nitrogen and stored at—80°C. Following tissues collection, the mice were euthanized by exsanguination. All animal procedures were approved by the Institutional Animal Care and Use Committee at the University of California, Davis.

### Protein synthesis measurements

Protein synthesis was measured *in vivo* in the WT mice using the SUnSET method as previously described (Goodman et al., 2011b). Exactly 30 min before the plantaris muscles were excised, mice were given an intraperitoneal injection of 0.04 μmol/g puromycin dissolved in 100 μl of phosphate buffered saline (PBS) (*n* = 5/group). Puromycin expression was analyzed by Western Blot as described below.

### mRNA expression analysis

Total RNA was extracted from powdered plantaris muscle using TRIzol reagent according to the manufacturer's instructions (Invitrogen). cDNA was then synthesized using a QuantiTech Reverse Transcription Kit (Qiagen) from one μg of total RNA. *MuRF1 and MAFbx g*ene expression was measured by quantitative PCR (qPCR) in WT mice following 1, 3, 7, and 14 days of FO (*n* = 7/group). qPCR was performed using *Power* SYBR® Green PCR Master Mix (Life Technologies) on an ABI 7900HT thermocycler. Cycling conditions were one cycle at 94°C for 10 min followed by forty cycles at 94°C for 30 s, 59°C for 30 s, and 72°C for 30 s. Each sample was run in triplicate. Sequences of the mouse forward and reverse primers are as follows: MuRF1 forward: 5'-GCTGGTGGAAAACATCATTGACAT-3'; reverse: 5'-CATCGGGTGGCTGCCTTT-3'; MAFbx forward: 5'-CTTTCAACAGACTGGACTTCTCGA-3'; reverse: 5'-CAGCTCCAACAGCCTTACTACGT-3'; FOXO1 forward: 5'-TTCCTTCATTCTGCACACGA-3'; reverse: 5'-GTCCTACGCCGACCTCATC-3'; FOXO3a forward: 5'-CAGGCTCCTCACTGTATTCAGCTA-3'; reverse: 5'-CATTGAACATGTCCAGGTCCAA-3'; GAPDH forward: 5'- CCAGCCTCGTCCCGTAGAC-3'; reverse: 5'- ATGGCAACAATCTCCACTTTGC-3'. All data was normalized to GAPDH expression.

### Proteasome activity

20S and 26S β5 proteasome activity was measured as previously described (Gomes et al., 2012). Briefly, proteasomes were collected in the supernatant after 30 min centrifugation at 12,000 *g* following homogenization in 300 μl of buffer containing 50 mM Tris, 150 mM NaCl, 5 mM MgCl2, 1 mM EDTA, and 0.5 mM DTT at pH 7.5. The chymotrypsin (β5)-like activities were assayed using 10 μg of protein and the fluorescently tagged substrate SUC-LLVY-AMC (Bachem). Both assays were carried out in a total volume of 100 μl. The 26S ATP-dependent assay was performed in homogenization buffer with the addition of 100 μM ATP. The 20S ATP-independent assay was carried out in assay buffer containing 25 mM HEPES, 0.5 mM EDTA, and 0.001% SDS (pH 7.5). Each assay was conducted in the absence and presence of the proteasome inhibitor Bortezomib at a final concentration of 2 mM. The activity of the 20S and 26S proteasome was measured by calculating the difference between fluorescence units recorded with or without the inhibitor in the reaction medium. Released AMC was measured using a Fluoroskan Ascent fluorometer (Thermo Electron) at an excitation wavelength of 390 nm and an emission wavelength of 460 nm. Fluorescence was measured at 15-min intervals for 2 h. All assays were linear in this range and each sample was assayed in triplicate.

### Western blotting

Frozen plantaris muscles from control and FO mice were homogenized in proteasome assay lysis buffer (50 mM Tris, 150 mM NaCl, 5 mM MgCl2, 1 mM EDTA, and 0.5 mM DTT at pH 7.5). The supernatant was collected following centrifugation at 12,000 *g* for 30 min and protein concentrations were determined in triplicate using the Bradford method (Bio-Rad). Ten to twenty micrograms of protein was subjected to SDS-PAGE on 10% acrylamide gels and transferred to polyvinylidene diflouride (PVDF) membrane. Membranes were blocked in 3% nonfat dairy milk in Tris-buffered saline with 0.1% Tween-20 added (TBST) or 1% pigskin gelatin for 1 h and then probed with primary antibody overnight at 4°C. Puromycin (Millipore), BiP (BD Biosciences), PDI (Cell Signaling), and CHOP (Cell Signaling) were used at a concentration of 1:1000. The next day, membranes were washed and incubated with HRP conjugated secondary antibodies at 1:10,000 for 1 h at room temperature. Immobilon Western Chemiluminescent HRP substrate (Millipore) was then added to the membranes. Image acquisition and band quantification was performed using the ChemiDoc™ MP System and Image Lab 5.0 software (Biorad).

### Statistics

Results are presented as mean ± standard deviation (*SD*) unless otherwise indicated. The data was analyzed by One-Way ANOVA or by Student's *t*-test (Sigma Stat). Tukey's *post-hoc* analysis was used to determine differences when interactions existed. Statistical significance was set at *p* < 0.05.

## Results

**Figure 1:**
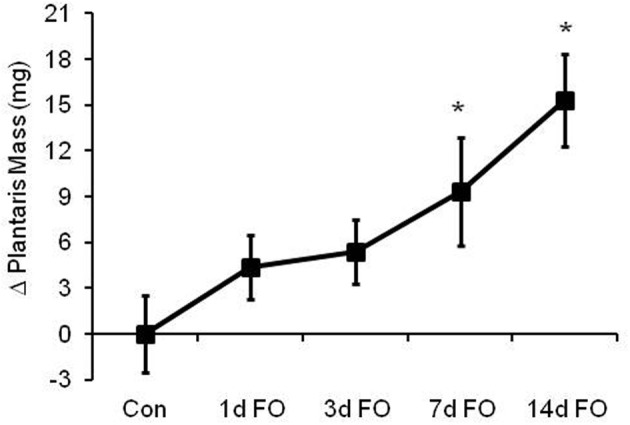
**Time course of load-induced growth in wild type (WT) mice following functional overload (FO)**. Growth of the plantaris muscle was calculated after 1, 3, 7, and 14 days of FO and expressed as a change in mass relative to the control group. Data are expressed as mean ± SD (*n* = 8/group). ^*^P < 0.05 vs. control.

To determine the extent to which the ubiquitin proteasome system (UPS) is activated during a model of load-induced muscle growth, male C57BL/6 mice were subjected to bilateral functional overload for 1, 3, 7, or 14 days. As shown in Figure 1, the response of the plantaris to an increase in load was a swift and steady increase in size over 14 days. Significant increases in mass of 43 and 65% were observed after 7 and 14 days, respectively.

**Figure 2:**
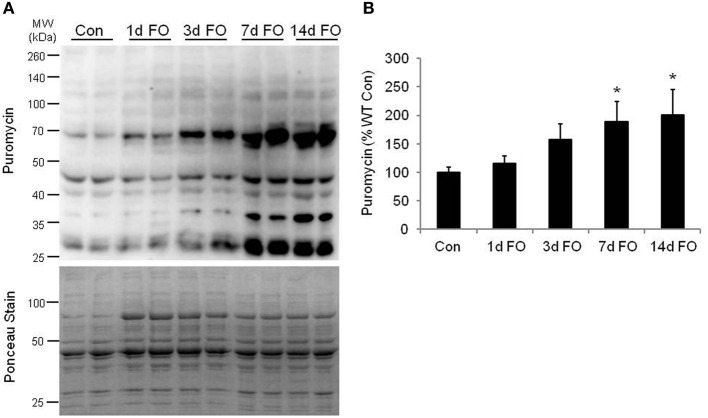
**Measurement of protein synthesis using the SUnSET method following functional overload (FO). (A)** Representative image of western blot analysis for puromycin following no treatment (Con) and FO for 1, 3, 7, or 14 days in WT mice. The corresponding ponceau stain was used to verify equal loading of proteins. **(B)** Quantification of the puromycin western blots. Puromycin values are expressed as a percentage of the control muscles value (mean ± *SD, n* = 5/group). ^*^P < 0.05 vs. control puromycin expression.

Given the significant hypertrophy of the plantaris muscle, we next looked at changes in protein synthesis in the plantaris muscle over a 14-day period of FO. Protein synthesis was measured *in vivo* using the SUrface SEnsing of Translation (SUnSET) method, a nonradioactive technique in which changes in the rate of protein synthesis are reflected by the amount of puromycin that is incorporated into newly synthesized proteins (Schmidt et al., 2009; Goodman et al., 2011b). Using this method, we found that protein synthesis increased by 58% at 3 days and was significantly elevated from 7 to 14 days of FO, reaching a level that was 100% above control within the first 14 days of FO (Figure 2).

**Figure 3:**
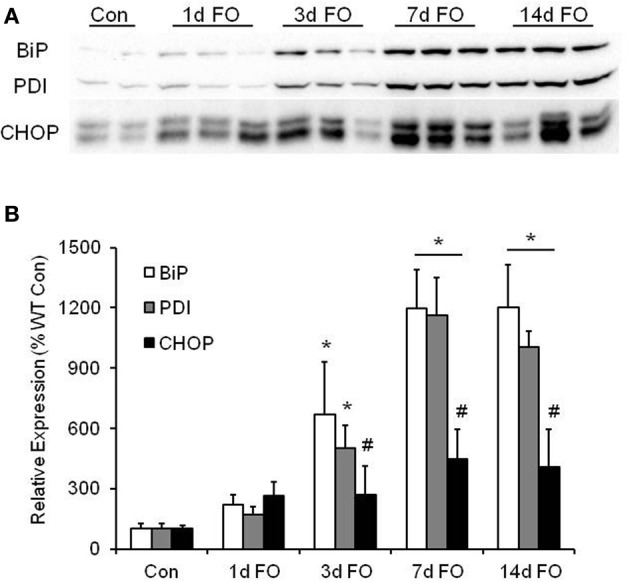
**Markers of endoplasmic reticulum (ER) stress are higher in the plantaris muscle following functional overload (FO). (A)** Representative western blot of ER stress markers BiP, PDI, and CHOP in WT mice after no treatment (Con) and 1, 3, 7, and 14 days of FO. **(B)** Quantification of the western blots for BiP (open bars), PDI (gray bars), and CHOP (black bars). A ponceau stain of the membrane was used to normalize protein expression. Data are expressed as a percentage of the respective control value for each protein (mean ± *SD, n* = 4–6/group). ^*^P < 0.05 vs. control expression for each protein, ^#^*P* < 0.05 CHOP vs. BiP, or PDI at given time point.

Increases in ER stress can occur during high rates of protein synthesis (Rayavarapu et al., 2012). Considering that FO produces significant muscle hypertrophy, we investigated the expression of various ER stress markers in the plantaris muscle following FO. As shown in Figure 3, we found significant increases in BiP and PDI expression beginning at 3 days post FO, with the largest increase seen after 7 days of FO. The maladaptive ER stress marker CHOP was also found to increase over the 14 days following FO, however, the relative increase was significantly lower than that observed for BiP and PDI (Figure 3B).

**Figure 4:**
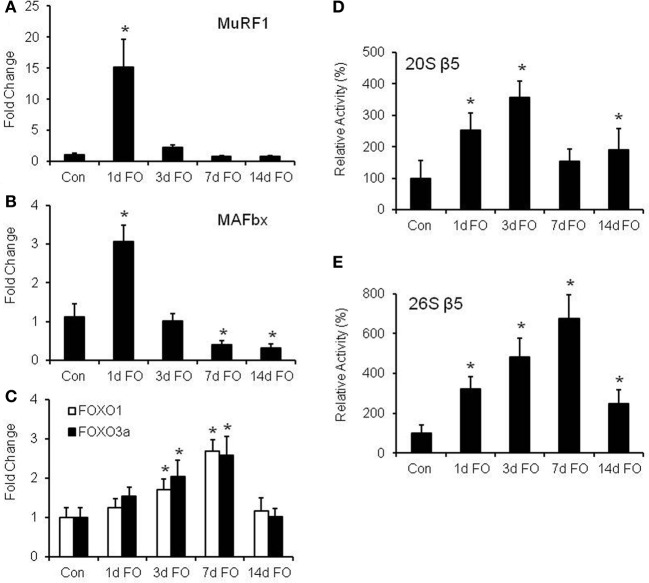
**Functional overload (FO) induces a proteolytic response in the plantaris muscle**. mRNA expression of **(A)** MuRF1, **(B)** MAFbx, and **(C)** FOXO1, and FOXO3a was measured in WT mice following no treatment (Con) and FO for 1, 3, 7, or 14 days. Expression values were normalized to GAPDH and are expressed as a fold change (mean ± *SD*) relative to control (*n* = 7/group) ^*^P < 0.05 vs. control mRNA expression. Activity of the β5 subunit of the 20S **(D)** and 26S **(E)** proteasome was assessed by fluorometric assay in WT mice following no treatment (Con) and FO for 1, 3, 7, or 14 days. Data is expressed as a percentage of the control value (mean ± *SD, n* = 5–8/group). ^*^P < 0.05 vs. control proteolytic activity.

In humans, resistance exercise has been shown to increase protein degradation (Phillips et al., 1997), but it is unclear whether this breakdown is related to upregulation of MuRF1 and MAFbx expression or alterations in proteasome activity. Thus, we measured the time course of MuRF1 and MAFbx expression and the chymotrypsin-like (β5) proteasome activities in WT mice following FO. In addition, the time course of FOXO1 and FOXO3a expression was measured since they are known transcriptional regulators of MuRF1 and MAFbx under atrophy conditions (Sandri et al., 2004; Waddell et al., 2008). Expression of both MuRF1 and MAFbx was found to increase significantly after one day of FO, but then return to control levels by 3 days post FO (Figures 4A,B). After 3 days of FO, gene expression was suppressed below control levels, with significant reductions in MAFbx expression occurring after 7 and 14 days of FO (Figure 4B). The rapid increase in MuRF1 and MAFbx expression was mirrored by significant increases in 20S and 26S β5 proteasome activity after 1 day of FO, but unlike MuRF1 and MAFbx, proteasome activity remained elevated throughout the 14 days of chronic loading (Figures 4D,E). Peak activity for the 20S proteasome was found to occur at 3 days post FO, while peak activity for the 26S proteasome was found at 7 days post FO. Surprisingly, the pattern of FOXO1 and FOXO3a expression was more similar to that of the 26S β5 proteasome rather than MuRF1 and MAFbx expression. Significant increases in FOXO1 and FOXO3a expression did not occur until 3 days post FO, after which expression of both genes continued to increase through 7 days of FO before returning to baseline levels at 14 days post FO (Figure 4C).

**Figure 5:**
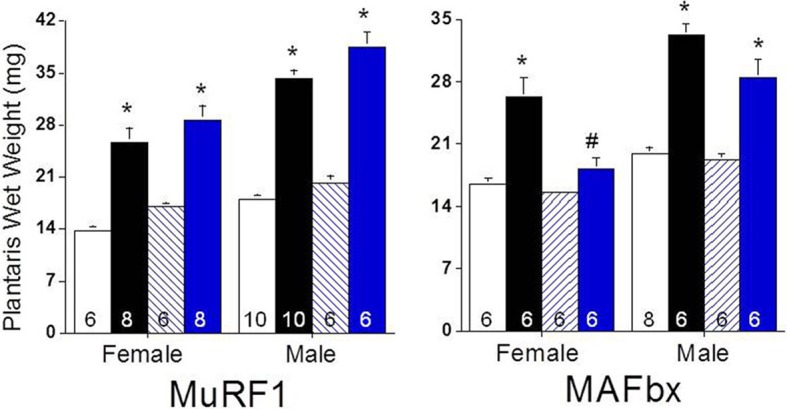
**Response of MuRF1 and MAFbx KO mice to functional overload (FO)**. Growth of the plantaris muscle was calculated after 14 days of FO in male and female WT, MuRF1 KO, and MAFbx KO mice and expressed as absolute wet weight (mg). Measurements were made in untreated control [white (WT) or blue hatched (KO) bars] and overloaded [black (WT) and blue (KO) bars] muscles. Data are expressed as mean ± s.e.m and group size is indicated in each bar. ^*^P < 0.05 vs. control; ^#^*P* < 0.05 vs. WT FO.

Given the changes in MuRF1 and MAFbx expression and proteasome activity following functional overload, we then asked if deletion of MuRF1 or MAFbx compromised muscle growth. Fourteen days of overload produced significant growth of the plantaris in both female and male MuRF1 KO mice, which was similar to that observed in WT mice of both genders (Figure 5). This result is comparable to what has been previously reported for male MuRF1 KO mice (Hwee et al., 2013). In contrast, the deletion of MAFbx appeared to have a significant effect on load-induced growth, especially in female mice (Figure 5). In response to overload, a significant increase in plantaris mass was measured in male MAFbx KO mice, with the mean increase in mass being slightly less and more variable in the MAFbx KO (range of mass: 20–34 mg) compared to the WT (range of mass: 29–37 mg) mice. In female mice, however, MAFbx KO mice showed no significant growth in response to FO, which differed significantly from what was observed in the WT mice (Figure 5).

**Figure 6:**
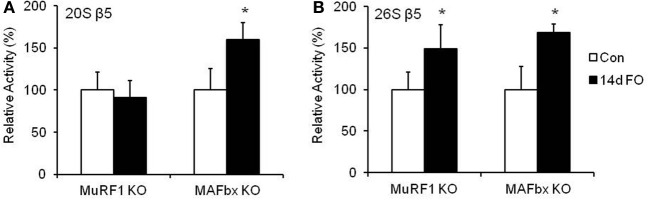
**Proteasome activity increases in MuRF1 and MAFbx null (KO) mice after 14 days of functional overload (FO)**. Proteolytic activity of the β5 subunit of the 20S **(A)** and 26S **(B)** proteasome was assessed by fluorometric assay in MuRF1 and MAFbx KO mice after no treatment (Con, open bars) or 14 days of functional overload (14d FO, black bars). Data are expressed as a percentage of each respective control value (mean ± *SD, n* = 4–6/group). ^*^P < 0.05 vs. control.

The decrease in muscle growth did not appear to be due to an inability to activate the proteasome, as both MuRF1 and MAFbx KO mice had similar increases in 26S β5 proteasome activity following 14 days of FO (Figure 6). Since we did not have sufficient numbers of KO mice to collect FO data at 3 and 7 days, we do not know whether proteasome activity in the KO mice increased to the same extent as the WT mice.

## Discussion

Proteolysis is essential for normal muscle function and routine protein turnover. Most cellular proteins are degraded by the UPS (Rock et al., 1994; Mitch and Goldberg, 1996), a highly selective system that targets proteins for breakdown via the addition of a polyubiquitin chain. The coordinated effort of three groups of enzymes, termed E1, E2, and E3s, results in the attachment of ubiquitin to a substrate protein, with multiple lysine 48-linked ubiquitin molecules serving as a signal for that protein to be degraded by the 26S proteasome (Chau et al., 1989). Both MuRF1 and MAFbx have been identified as muscle-specific E3 ubiquitin ligases, making them responsible for catalyzing the transfer of ubiquitin from the E2 enzyme to the substrate protein. In skeletal muscle, increases in proteasome activity are generally associated with muscle atrophy, a process that is characterized by the induction of MuRF1 and MAFbx (Auclair et al., 1997; Hobler et al., 1999; Bodine et al., 2001a; Gomes et al., 2001). However, little is known about the role of the UPS during muscle growth and whether MuRF1 and/or MAFbx are required for muscle hypertrophy. Thus, the purpose of this study was to examine the time course of MuRF1 and MAFbx expression along with proteasome activity in a model of load-induced muscle growth, and to determine if MuRF1 and MAFbx KO mice show an attenuated growth response following 14 days of functional overload (FO).

Functional overload is a commonly used model for studying muscle growth in rodents and results in rapid and robust increases in muscle mass as a result of chronic overload. Hypertrophy in this model is marked by significant increases in protein synthesis, which we confirmed in this study using the puromycin technique (Schmidt et al., 2009; Goodman et al., 2011b). Moreover, the increase in protein synthesis was closely matched to the increase in plantaris mass in the WT mice. During this period of elevated protein synthesis, significant increases in the expression of the ER chaperone proteins BiP and PDI were also observed. An increase in BiP and PDI expression might be predicted, as an elevated rate of protein synthesis would increase the protein handling responsibilities of the endoplasmic reticulum (ER). An increase in BiP and PDI enhances the protein folding capabilities of the ER (Rayavarapu et al., 2012) and would help reduce the number of misfolded proteins and keep ER stress at a minimum. An accumulation of misfolded proteins can cause the ER to activate apoptosis signaling through an increase in the expression of CHOP (Fu et al., 2008). Although FO did result in an increase in CHOP expression, the relative increase in expression was significantly lower than the increase in BiP and PDI expression, suggesting that the ER was able to implement an adaptive response to the influx of newly synthesized proteins, which ultimately get incorporated into the myofibers resulting in increases in myofiber cross-sectional area and force capacity.

A novel finding in this study was that both 20S and 26S β5 proteasome activity was increased throughout the 14 day overload period, indicating that increased loading can result in the activation of machinery involved in protein breakdown. The increase in proteasome activity occurred within the first 24 h of overload and increased to a level (4–6-fold) that was much greater than what we have observed during denervation-induced atrophy (<2-fold) (Gomes et al., 2012). Interestingly, the peak in 26S β5 activity occurred at 7 days, a time when protein synthesis was found to be significantly elevated. This finding is similar to a study by Miyazaki et al. in which protein synthesis and protein degradation rates were both found to peak at 7 days after FO (Miyazaki et al., 2011). However, it is important to note that the largest gains in muscle mass occurred between 7 and 14 days post FO, which was the time period in which protein synthesis rates were rising and proteasome activity was beginning to decrease.

An increase in MuRF1 and MAFbx expression is generally assumed to lead to an increase in proteasome activity, as a greater quantity of ubiquitin ligases should increase the number of polyubiquitinated proteins inside the cell. However, we show here that under growth conditions, proteasome activity remained elevated for a much longer time period than did the induction of MuRF1 and MAFbx. In fact, significant increases in MuRF1 and MAFbx expression were measured only at day one of FO, and by day 3 of FO, their expression had returned to baseline levels and then were suppressed below baseline levels. Our finding that MuRF1 expression is only increased at 1 day post FO differs slightly from a study by Marino et al., in which MuRF1 was found to be increased after 3 days of FO (Marino et al., 2008). However, similar results were found when comparing MAFbx expression, as we also found no induction of MAFbx at 3 days after FO followed by a significant decrease in MAFbx expression at 7 and 14 days (Marino et al., 2008). In the majority of human studies that examined proteolytic activity after an acute bout of resistance exercise, MuRF1, but not MAFbx expression has been shown to increase transiently after the exercise bout (Yang et al., 2006; Louis et al., 2007; Murton et al., 2008). However, chronic resistance training in rats resulted in decreased MuRF1 and MAFbx expression, which may be in line with this decreased expression we saw at 7 and 14 days following FO (Zanchi et al., 2009).

Our data show that MuRF1 and MAFbx are not always good markers of proteasome activity. The apparent disconnect between MuRF1 and MAFbx expression and proteasome activity has been previously observed. In a study by Vary et al., acute alcohol intoxication increased MuRF1 and MAFbx expression, but did not increase skeletal muscle proteolysis (Vary et al., 2008). Similarly, we have shown that 14 days of glucocorticoid treatment did not result in an increase in activity for any of the three catalytic subunits of the proteasome despite significant upregulation of MuRF1 and MAFbx expression (Baehr et al., 2011). Conversely, when mice were allowed to recover following 7 days of hindlimb unloading, MuRF1 and MAFbx expression was not increased at any of the time points analyzed, but 20S β5 proteasome activity was significantly increased on the first day of recovery (Lang et al., 2012). Lastly, under denervation conditions, the lack of MuRF1 resulted in greater activation of the proteasome, not less (Gomes et al., 2012).

Under atrophy conditions, the FOXOs are often implicated in the induction of MuRF1 and MAFbx, but our results clearly indicate that this is not the case in the functional overload model, as FOXO expression did not increase until after MuRF1 and MAFbx expression had returned to baseline levels. The largest increase in FOXO1 and FOXO3a expression was found to occur after 7 days of functional overload, which is consistent with the findings of Goodman et al. (2011a) who showed that both total protein and phosphorylation levels of FOXO1 and FOXO3a were significantly elevated at 7 days of FO. Our results suggest that the FOXOs may be mediating protein degradation independently of MuRF1 and MAFbx, and may be at least partially responsible for the observed increase in proteasome activity. More work is needed to determine the role of the FOXOs in regulating the ubiquitin proteasome system during skeletal muscle growth.

Mechanical loading has been shown to initiate an inflammatory response and a number of cytokines have been reported to increase during muscle hypertrophy (Huey et al., 2007). One cytokine in particular that was reported to be elevated early after FO was TNFα (Huey et al., 2007). Circulating levels of TNFα can lead to increases in both MuRF1 and MAFbx expression (Li et al., 2005; Adams et al., 2008), so it is possible that the short-lived increase in expression seen in this study was directly related to muscle inflammation. While an inflammatory response appears to be required for normal growth following FO (Marino et al., 2008), it is unclear whether induction of MuRF1 and MAFbx is critical in this response. Our results indicate that MAFbx, but not MuRF1, may be necessary for normal remodeling and growth, as the MAFbx KO mice had an attenuated growth response (especially among the female animals), whereas the MuRF1 KO mice showed no deficiencies in their ability to hypertrophy.

In skeletal muscle, a few targets of MAFbx have been identified, including eIF3f (Lagirand-Cantaloube et al., 2008), MyoD (Tintignac et al., 2005), and myogenin (Jogo et al., 2009). These targets are generally associated with protein synthesis (eIF3f), satellite cell proliferation (MyoD), and muscle-specific gene transcription (MyoD, myogenin), all of which are important for muscle hypertrophy (Ishido et al., 2004; Baar et al., 2006). In addition, recent *in vitro* work by Lokireddy et al. revealed that MAFbx preferentially degrades sarcomeric proteins following myostatin treatment, with myosin heavy chain, myosin light chain, desmin, and vimentin identified as targets of MAFbx ubiquitination (Lokireddy et al., 2011a,b). Thus, even though it appears that the lack of MAFbx should promote muscle growth, the inability to turnover key sarcomeric proteins, such as myosin heavy chain, during the remodeling process could explain why the growth response was impaired in the MAFbx KO mice. Furthermore, while MAFbx KO mice have been shown to spare muscle mass following denervation (Bodine et al., 2001a), histological analysis of denervated MAFbx muscles has revealed dystrophic and necrotic fibers. Consequently, it appears that MAFbx may be required for the proper remodeling of muscle fibers under growth and atrophy conditions. The explanation for the finding that the loss of MAFbx had a greater effect on load-induced growth in female vs. male mice is not clearly evident. In previous experiments that have examined the response of MAFbx KO mice to triggers of muscle atrophy, we have observed no gender-based differences.

Similar to MAFbx, MuRF1 has been reported to interact and ubiquitinate myofibrillar proteins (Cohen et al., 2009), suggesting that MuRF1 also plays a role in regulating protein turnover. However, given the normal hypertrophic response to FO in the MuRF1 KO mice, it seems that MuRF1 is not essential for muscle growth. Considering that protein synthesis is higher in MuRF1 KO mice under atrophy conditions (Koyama et al., 2008; Baehr et al., 2011), it may be that the major role of MuRF1 in skeletal muscle is to suppress protein synthesis. Thus, deletion of MuRF1 is advantageous to muscle growth and consequently, the MuRF1 KO mice maintain an ability to hypertrophy throughout their lifetime. The different phenotypes in the MuRF1 and MAFbx KO mice suggest that the two E3 ligases have different physiological substrates. Clearly more research is needed to determine the physiological targets of both MuRF1 and MAFbx in skeletal muscle.

In summary, our results indicate that muscle hypertrophy is associated with increases in both protein synthesis and degradation. The increase in degradation is the result of activation of the UPS, and proteasome activity remains elevated even after MuRF1 and MAFbx expression has returned to baseline levels. Interestingly, MuRF1 and MAFbx expression become suppressed below baseline even though FOXO1 and FOXO3a expression are elevated. The loss of MuRF1 or MAFbx does not appear to suppress the increase in proteasome activity in response to chronic increases in load; however, the loss of MAFbx does appear to negatively impact the remodeling process that occurs during growth. These findings highlight the need for a better understanding of the roles of MuRF1 and MAFbx in the function of skeletal muscle, which will require identification of their *in vivo* substrates.

### Conflict of interest statement

The authors declare that the research was conducted in the absence of any commercial or financial relationships that could be construed as a potential conflict of interest.
